# Transcriptionally Active Lung Microbiome and Its Association with Bacterial Biomass and Host Inflammatory Status

**DOI:** 10.1128/mSystems.00199-18

**Published:** 2018-10-30

**Authors:** Lili Ren, Rongbao Zhang, Jian Rao, Yan Xiao, Zhao Zhang, Bin Yang, Depan Cao, Hui Zhong, Pu Ning, Ying Shang, Mingkun Li, Zhancheng Gao, Jianwei Wang

**Affiliations:** a MOH Key Laboratory of Systems Biology of Pathogens and Christophe Mérieux Laboratory, Institute of Pathogen Biology, Chinese Academy of Medical Sciences & Peking Union Medical College, Beijing, China; b Department of Respiratory and Critical Care Medicine, Peking University People’s Hospital, Beijing, China; c Key Laboratory of Genomic and Precision Medicine, Beijing Institute of Genomics, Chinese Academy of Sciences, Beijing, China; d Fondation Mérieux, Lyon, France; e Center for Excellence in Animal Evolution and Genetics, Chinese Academy of Sciences, Kunming, China; Pacific Northwest National Laboratory

**Keywords:** airborne microorganisms, bacterial biomass, lung microbiome, lung microbiota, metatranscriptome, microbial communities

## Abstract

Alteration of the lung microbiome has been observed in several respiratory tract diseases. However, most previous studies were based on 16S ribosomal RNA and shotgun metagenome sequencing; the viability and functional activity of the microbiome, as well as its interaction with host immune systems, have not been well studied. To characterize the active lung microbiome and its associations with host immune response and clinical features, we applied metatranscriptome sequencing to bronchoalveolar lavage fluid (BALF) samples from 25 patients with chronic obstructive pulmonary disease (COPD) and from nine control cases without known pulmonary disease. Community structure analyses revealed three distinct microbial compositions, which were significantly correlated with bacterial biomass, human Th17 immune response, and COPD exacerbation frequency. Specifically, samples with transcriptionally active *Streptococcus*, *Rothia*, or *Pseudomonas* had bacterial loads 16 times higher than samples enriched for *Escherichia* and *Ralstonia*. These high-bacterial-load samples also tended to undergo a stronger Th17 immune response. Furthermore, an increased proportion of lymphocytes was found in samples with active *Pseudomonas*. In addition, COPD patients with active *Streptococcus* or *Rothia* infections tended to have lower rates of exacerbations than patients with active *Pseudomonas* and patients with lower bacterial biomass. Our results support the idea of a stratified structure of the active lung microbiome and a significant host-microbe interaction. We speculate that diverse lung microbiomes exist in the population and that their presence and activities could either influence or reflect different aspects of lung health.

**IMPORTANCE** Recent studies of the microbiome proposed that resident microbes play a beneficial role in maintaining human health. Although lower respiratory tract disease is a leading cause of sickness and mortality, how the lung microbiome interacts with human health remains largely unknown. Here we assessed the association between the lung microbiome and host gene expression, cytokine concentration, and over 20 clinical features. Intriguingly, we found a stratified structure of the active lung microbiome which was significantly associated with bacterial biomass, lymphocyte proportion, human Th17 immune response, and COPD exacerbation frequency. These observations suggest that the microbiome plays a significant role in lung homeostasis. Not only microbial composition but also active functional elements and host immunity characteristics differed among different individuals. Such diversity may partially account for the variation in susceptibility to particular diseases.

## Abstract (precis)

Recent studies of the microbiome proposed that resident microbes play a beneficial role in maintaining human health. Although lower respiratory tract disease is a leading cause of sickness and mortality, how the lung microbiome interacts with human health remains largely unknown. Here we assessed the association between the lung microbiome and host gene expression, cytokine concentration, and over 20 clinical features. Intriguingly, we found a stratified structure of the active lung microbiome which was significantly associated with bacterial biomass, lymphocyte proportion, human Th17 immune response, and COPD exacerbation frequency. These observations suggest that the microbiome plays a significant role in lung homeostasis. Not only microbial composition but also active functional elements and host immunity characteristics differed among different individuals. Such diversity may partially account for the variation in susceptibility to particular diseases.

## INTRODUCTION

Investigation of the lung microbiome is a relatively young field; however, there has been remarkable progress in understanding the composition and function of the lung microbiome in the last few years (1). Dickson and colleagues have described an adapted island model for the lung microbiome (2), in which lung microbes are largely derived from the upper respiratory tract and oral cavity (UO) through microaspiration and mucosal dispersion. Simultaneously, microbes in the lung are eliminated by mucociliary clearance, cough, and host immune defenses, while reproduction and growth of the microbes are determined by regional conditions in the lung. Alterations of the lung microbiome have been observed in many respiratory diseases, including chronic obstructive pulmonary disease (COPD), asthma, and cystic fibrosis, but associations with clinical features and interactions with host genes are largely unknown (3–5).

Sequencing of 16S rRNA gene amplicons is a convenient method to characterize the structure of the microbial community (6, 7). However, information provided by this method is limited due to its narrow detection spectrum (bacteria), low resolution (genus), and lack of direct insights into the viability and functional activity of the microbiome. An alternative approach is shotgun metagenome sequencing, which has been used to reveal the composition of the lung microbiome with a higher resolution and wider detection spectrum (8, 9); however, recent studies proposed that expression of microbial genes could significantly change without large alterations in overall community structure (10, 11), which emphasize the importance of investigating the functional activity of the microbiome. Metatranscriptome sequencing can provide not only active microbiome profiles at high resolution but also the details of functional elements (10, 12). Moreover, it enables analysis of interactions between the microbiome and the host, as both microbial and human transcripts can be analyzed (13). Such studies on the lung microbiome are very limited thus far, and there is an urgent need to explore the active host-microbe interaction in the lung.

COPD, a chronic inflammatory disorder characterized by long-term poor airflow, has been predicted by the World Health Organization to become the third leading cause of death by 2030 (14). The lung microbiome changes dramatically in COPD patients during the exacerbation period (15–17). However, microbes associated with COPD and exacerbation are inconsistent among different studies (15, 17–22). In this study, we aimed to provide a comprehensive description of transcriptionally active microbes and their associations with host gene expression, cytosine concentration, and different clinical features, on the basis of metatranscriptome data.

## RESULTS

### Overview of the active lung microbiome.

The transcriptionally active microbiome was examined in the bronchoalveolar lavage fluid (BALF) samples of 25 COPD patients (during their stable period) and 9 non-COPD controls by metatranscriptome sequencing (demographic and clinical information are described in Table S1 in the supplemental material). After stringent quality control was performed, 76.7% (±11.5%) of the reads were mapped to the human genome. Among the reads mapping to archaea, bacteria, fungi, and viruses (ABFV), 92% could be assigned to a specific genus, and 60% could be assigned to a specific species or subspecies. Rarefaction analysis showed that the current sequencing depth (30 million reads per sample) enabled us to study most genera/species with read abundance (proportion of reads among all ABFV reads, which reflects the transcriptional activity of the genera/species) greater than 0.1% but not those with lower read abundance. Thus, our study focused only on microbes with read abundance of at least 1% in at least one sample.

10.1128/mSystems.00199-18.8 TABLE S1 Background information for the recruited cases with chronic obstructive pulmonary disease (COPD) and non-COPD controls (ANB). Download Table S1, XLSX file, 0.02 MB. Copyright © 2018 Ren et al. 2018 Ren et al. This content is distributed under the terms of the Creative Commons Attribution 4.0 International license.

**FIG 1:**
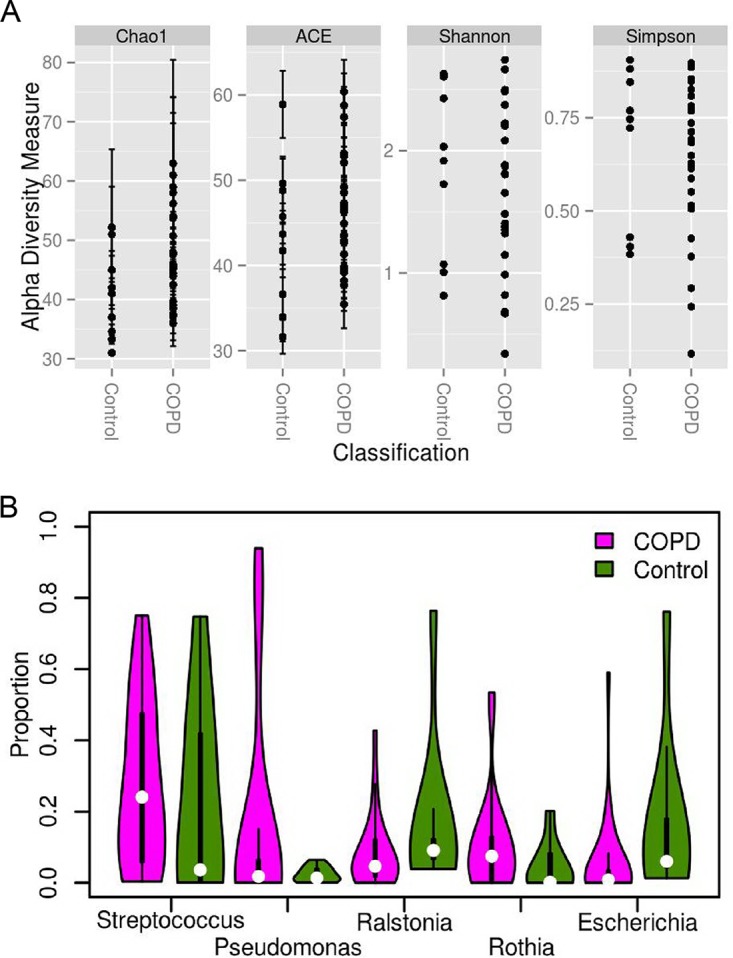
Diversity and composition of the lung microbiome at the genus level. (A) Alpha diversity values for COPD patients and non-COPD controls. (B) Violin plot of the active lung microbiome composition; only genera with a mean read abundance of at least 5% are shown; thickness indicates the density of the value, and each white dot indicates the median value.

Three phyla (*Proteobacteria*, *Firmicutes*, and *Actinobacteria*) were detected in all 34 samples and accounted for 91% of the total ABFV reads (see Fig. S1 in the supplemental material). At the genus level, *Streptococcus*, *Pseudomonas*, *Ralstonia*, *Escherichia*, and *Rothia* were the most abundant; those genera accounted for 66% of all ABFV reads and explained more than 70% of the variance among samples (Fig. 1). Eighty-seven species from 49 genera were identified. Among all genera, *Streptococcus* and *Pseudomonas* were most diverse, with 14 species and 11 species detected, respectively. Of note, the number of species identified was partially determined by the richness of diversity of sequences included in the reference database; thus, well-studied genera (such as *Streptococcus* and *Pseudomonas*) could be more diverse at the species level. However, only a limited number of species belonging to the same genus could be identified in any one sample.

10.1128/mSystems.00199-18.2 FIG S1 Diversity and composition of the lung microbiome. (A) Alpha diversity measurements of COPD and non-COPD controls at the phylum level. (B) Alpha diversity measurements of COPD patients and non-COPD controls at the species level. (C) Violin plot of the lung microbiome composition inferred from metatranscriptome data; only phyla with a mean read abundance of at least 1.5% are shown. (D) Violin plot of the lung microbiome composition inferred from metatranscriptome data; only species with a mean read abundance of at least 5% are shown. Download FIG S1, TIF file, 0.8 MB. Copyright © 2018 Ren et al. 2018 Ren et al. This content is distributed under the terms of the Creative Commons Attribution 4.0 International license.

No significant difference was found between COPD and non-COPD samples in terms of alpha diversities and microbial composition with our data (*P > *0.05 and *P > *0.05, respectively) (Fig. 1; see also Fig. S1). Several microbes showed marginal read abundance differences between the two groups. At the genus level, *Gemella* was observed in only 10 COPD samples and at very low read abundance (median = 0.03%; *P < *0.05, false-discovery rate [*q*]* *>* *0.1). At the species level, four low-read-abundance species (Prevotella enoeca, Neisseria gonorrhoeae, Bifidobacterium dentium, and Enterococcus cecorum) were enriched in COPD samples (*P < *0.05, *q *>* *0.1), and the first three species are known to inhabit the oral cavity and upper respiratory tract. Notably, the sample size in the study was relatively small, which provided us very limited power to detect differences; thus, we aimed to identify the features that are most closely associated with the active lung microbiome.

### Consistency between metatranscriptome sequencing results and 16S rRNA sequencing results.

**FIG 2:**
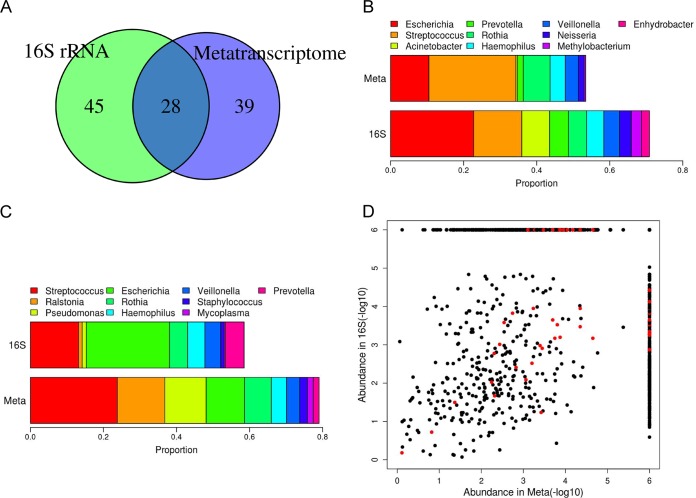
Comparison of microbiome composition between metatranscriptome data and 16S rRNA data. (A) Overlap of identified genera between two data sets. (B) The read abundance of the top 10 most abundant genera in 16S rRNA data in two data sets. (C) The read abundance of the top 10 most abundant genera in metatranscriptome data in two data sets. (D) Correlation of the read abundance of each genus between the two methods at the individual level. Red dots denote the genus detected in patient COPD38, who had the highest metatranscriptome-versus-16S rRNA correlation (rho = 0.99, *P* < 0.001). For display, an abundance of 0 was converted to 10^−6^.

16S rRNA sequencing data were successfully obtained for twenty samples (V3-V4 region, with at least 10,000 reads); 73 genera were identified, 28 of which were also discovered in the metatranscriptome data (Fig. 2A). Those 28 genera accounted for 83% of the 16S rRNA reads and 89% of the ABFV metatranscriptome reads, suggesting that the high-abundance genera could be faithfully identified by both methods. However, the abundances determined were not always comparable between the two methods (Fig. 2B and C); for example, *Acinetobacter* had a higher abundance in the 16S rRNA data, while *Ralstonia* and *Pseudomonas* were more highly enriched in the metatranscriptome data. The overall correlation coefficient for the read abundance of each genus estimated on the basis of these two methods was 0.326 (*P < *0.001), with the highest correlation coefficient being 0.99 in patient COPD38 (*P < *0.001) (Fig. 2D). Discrepancies between the two methods might reflect the different states of the microbes, which could be either active/viable (overrepresented in metatranscriptome data) or resting/suppressed (underrepresented in the metatranscriptome data). However, an alternative explanation could be that samples with low correlation coefficients had lower bacterial biomass and, hence, that their lung microbiomes were more likely to have been contaminated by reagents and/or the bronchoscope. This hypothesis was supported by the fact that correlation coefficients were positively correlated with bacterial biomass quantified by a 16S rRNA assay (*P = *0.02, rho = 0.560).

### Structure of the active lung microbiome.

**FIG 3:**
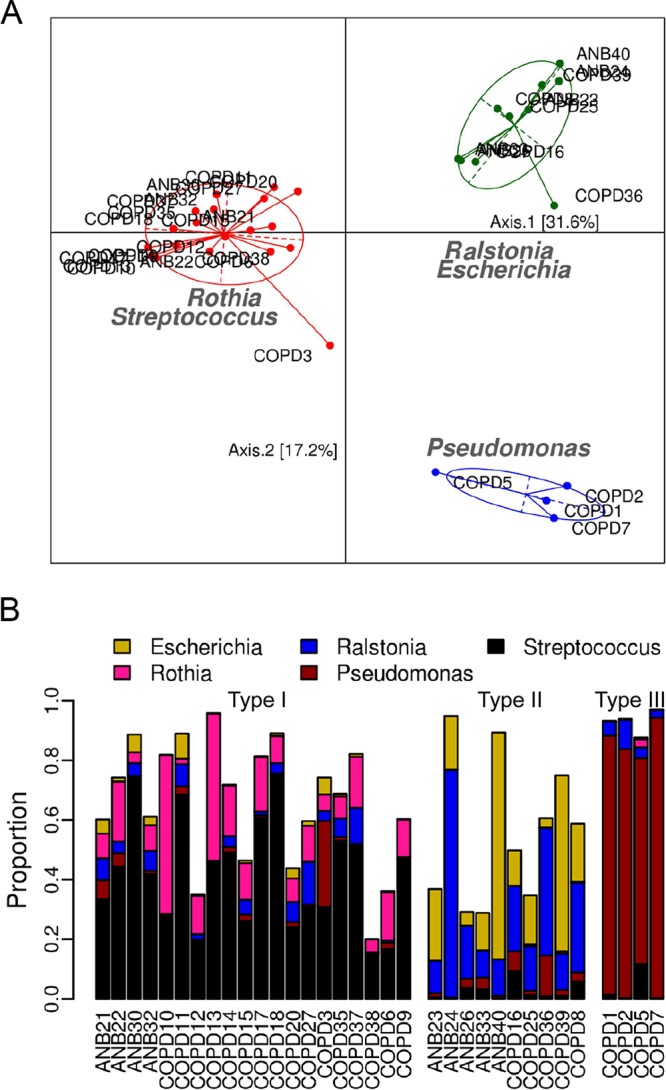
Structure of the lung microbiome at the genus level. (A) Principal-coordinate-analysis (PCoA) plot of the active lung microbiome inferred from metatranscriptome data. Core microbes are labeled on the plot, and the pairwise distance is represented by the Jensen-Shannon divergence (JSD) value. (B) Read abundance of the core microbes in different individuals; samples are ordered by the subgroups to which they belong.

Possible lung microbiome subgroups were investigated using two statistical methods (Dirichlet multinomial mixtures and partitioning around medoid clustering coupled with the heuristic Calinski-Harabasz index; see details in Text S1 in the supplemental material), which gave very similar results (only one sample was assigned differently at the phylum level). At the phylum level, samples were clustered into two subgroups, which were dominated by either *Proteobacteria* or *Actinobacteria* and *Firmicutes* (Fig. S2A and C). We found that the read abundances of the dominant (core) microbes (the definition and algorithm are described in Text S1) in the two subgroups were negatively correlated (*P < *0.01), whereas the read abundances of microbes within each subgroup were positively correlated (*P < *0.01). At the genus level, samples could be further classified into three subgroups (Fig. 3). Twenty samples were assigned to subgroup I, which was enriched for *Streptococcus* and *Rothia*; 10 samples were assigned to subgroup II, which was enriched for *Ralstonia and Escherichia*; and only 4 samples were assigned to subgroup III, whose microbiome was dominated by *Pseudomonas*. Core microbes of subgroup I were negatively correlated with those in subgroup II and subgroup III (*P < *0.01). Clustering at the species level was identical to that seen at the genus level (Fig. S2B and D).

10.1128/mSystems.00199-18.1 TEXT S1 Supplemental methods and results. Download Text S1, DOCX file, 0.04 MB. Copyright © 2018 Ren et al. 2018 Ren et al. This content is distributed under the terms of the Creative Commons Attribution 4.0 International license.

10.1128/mSystems.00199-18.3 FIG S2 Structure of the lung microbiome. (A and B) Principal-coordinate-analysis (PCoA) plot of the lung microbiome inferred from metatranscriptome data at the phylum level (A) and at the species level (B). Core microorganisms are labeled on the plot; the pairwise distance is represented by the Jensen-Shannon divergence (JSD) value. (C and D) Read abundance of core microorganisms in different individuals at the phylum level (C) and at the species level (D), ordered by the subgroups to which they belong. ANB26 was assigned to the green cluster by the Dirichlet multinomial mixtures method at the phylum level. Download FIG S2, TIF file, 0.8 MB. Copyright © 2018 Ren et al. 2018 Ren et al. This content is distributed under the terms of the Creative Commons Attribution 4.0 International license.

The significance of the group classification was further evaluated by three methods (average silhouette width, predictive strength, and simulation; see details in Text S1). Clustering at the phylum and genus levels was supported by all statistical metrics, while clustering at the species level had a relatively low predictive strength (0.679) (Fig. S3), suggesting that the clustering scheme is reliable.

10.1128/mSystems.00199-18.4 FIG S3 Assessment of clustering quality. Four quality indices are shown for the clustering at the phylum (A to D), genus (E to H), and species (I to L) levels. (A, E, and I) Calinski-Harabasz (CH). (B, F, and J) Average silhouette width (ASW). (C, G, and K) Prediction strength (PS). (D, H, and L) Simulation. A good clustering result should have the highest CH and ASW values, PS values of >0.8, and a simulation *P* value of <0.05. Download FIG S3, TIF file, 0.4 MB. Copyright © 2018 Ren et al. 2018 Ren et al. This content is distributed under the terms of the Creative Commons Attribution 4.0 International license.

### Associations between active lung microbiome and clinical features.

**FIG 4:**
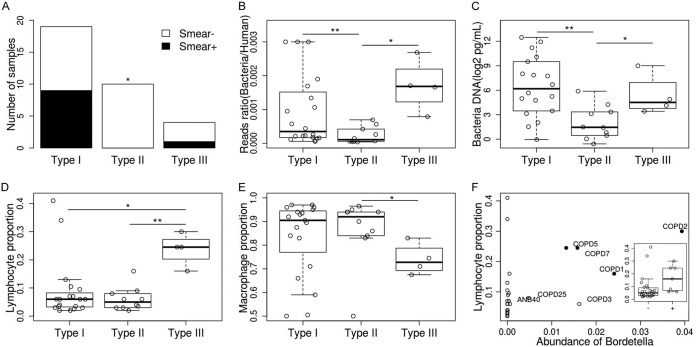
Association between the lung microbiome and clinical features. (A) Bacterial smear test results for different microbiome subgroup samples. (B) Ratio of bacteria reads to human reads. (C) Quantification of bacteria DNA. (D) Proportion of lymphocytes in BALF samples. (E) Proportion of macrophages in BALF samples. (F) Correlation between the proportion of lymphocytes and the read abundance of bacterial genus *Bordetella*; black dots denote samples in subgroup III. The box plot shows the lymphocyte proportion in *Bordetella*-positive samples and *Bordetella*-negative samples.

Associations between the structure of the active lung microbiome and 21 clinical features were investigated (Table 1). First, all samples in subgroup III were COPD patients, suggesting that this might be a COPD-specific group, though this hypothesis needs to be confirmed with a larger sample size. Second, all samples in subgroup II were negative in the bacteria smear test, which differs significantly from the results from the samples in subgroups I and III (*P < *0.05) (Fig. 4A). We hypothesized that this difference could be an indication of lower bacterial biomass for subgroup II, an interpretation that was supported by the observation that samples in subgroup II had the lowest ratio of bacterial reads to human reads (*P < *0.01, Fig. 4B). As the proportion of reads could have been biased by the amplification process during library preparation, the actual amount of bacterial DNA was further quantified by a 16S rRNA assay. The median amounts of bacteria DNA in the subgroup I and III samples were 28-fold and 9-fold higher, respectively, than the bacterial DNA amounts in subgroup II samples (77.2 pg/ml and 25.6* *pg/ml versus 2.7 pg/ml) (*P < *0.05) (Fig. 4C). Of note, Salter and colleagues proposed that contamination from laboratory reagents critically impacted results obtained from low-microbial-biomass samples, and both *Escherichia* and *Ralstonia* were on their list of contaminant genera (23). To examine the possibility of contamination, we collected two saline samples (washing through different bronchoscopes before real samples were collected) and prepared the sequencing library following the protocol that had been used with the negative controls. We found that the major compositions were similar, and both *Escherichia* and *Ralstonia* were identified in two negative controls (with read abundances of 35% and 0.1%, respectively) (Fig. S4). Thus, the possibility of contamination is high for this subgroup, and the high read abundance of *Escherichia* and *Ralstonia* may reflect only the background noise introduced from reagents and/or the bronchoscope. In addition, the lymphocyte proportion for the subgroup III samples was significantly higher than that for other subgroups (*P < *0.05), while the macrophage proportion was lower in this subgroup (*P < *0.05) (Fig. 4D and E). We further found that the lymphocyte proportion was positively correlated with the relative read abundance of *Bordetella* (mostly Bordetella pertussis) (*rho *=* *0.501, *P < *0.01, Fig. 4F). No correlation was found between the subgroups and the severity of COPD, smoking, or use of inhaled corticosteroids, bronchodilators, or other factors (Table 1).

**TABLE 1:** Tests of the association between the structure of the active lung microbiome and clinical features^*a*^

Phenotype	Range or results^*k*^	*P* value
COPD	{Yes, no}	0.129^*h*^
Smoking category	{Smoker, quit, never}	0.338^*h*^
Smoking amount, range^*b*^	[0, 60]	0.229^*i*^
Inflammation^*c*^	{Yes, no, unclear}	0.487^*h*^
Gender	{Male, female}	0.378^*h*^
Location	{Left lower lobe, left lingular lobe, right middle lobe}	0.631^*h*^
Age range (yrs)	[28, 83]	0.355^*i*^
Smear test	{Positive, negative}	0.019^*h*^
Inhaled corticosteroids	{Yes, no}	0.731^*h*^
Bronchodilators	{Yes, no}	0.553^*h*^
Exacerbation time^*d*^	[0, 3]	0.819^*i*^
Macrophage^*e*^ (%)	[0, 100%]	0.153^*i*^
Lymphocyte (%)	[0, 100%]	0.021^*i*^
Neutrophil (%)	[0, 100%]	0.896^*i*^
FEV1	[28.3, 99.3]	0.593^*i*^
FEV1FVC	[34.4, 70.13]	0.650^*i*^
RV/TLC	[5.2, 88.6]	0.476^*i*^
CAT	[2, 23]	0.057^*i*^
mMRC	{0, 1, 2, 3}	0.904^*h*^
Severity score (GOLD)^*f*^	{1, 2, 3, 4}	0.3628^*h*^
Exacerbation frequency^*g*^	{0.4, 2, 3.5}	1.3e−5^*j*^

a Use of inhaled corticosteroids and bronchodilators in the previous 3 months prior to the bronchoscope examination was considered. Antibiotics were not used at least 8 weeks preceding the bronchoscopy. CAT, COPD assessment test; FEV1, median forced expiratory volume in 1 s; FVC, forced vital capacity; mMRC, modified Medical Research Council dyspnea scale; RV, residual volume; TLC, total lung capacity;

b Data represent numbers of packs of cigarettes smoked per year.

c Inflammation status was judged by clinician during bronchoscopy.

d Data represent numbers of exacerbations during the year preceding the bronchoscopy.

e Cells in the BALF were collected and stained with Wright Giemsa’s stain, and cells were counted under a microscope.

f GOLD, Global Initiative for Obstructive Lung Disease criteria.

g Data represent frequencies of exacerbations for COPD patients in the previous 4 years (2014 to 2018) after the collection of BALF samples.

h For discrete data, the contingency table was created and the Fisher exact test was used for the significance test; thus, we were testing whether a specific classification (e.g., male or female) was associated with one of the three active lung microbiome subgroups.

i For continuous data, the Kruskal-Wallis rank sum test was applied; thus, we were testing whether a given feature was different among three different active lung microbiome subgroups.

j For frequency data, the chi-square test was used for the significance test; thus, we were testing whether the events were randomly distributed in different active lung microbiome subgroups.

k Braces mean all possible elements are given here (discrete variable). Square brackets mean a range is given here, e.g., from 0 to 60 (including 0 and 60)(continuous variable).

10.1128/mSystems.00199-18.5 FIG S4 The microbiome composition for negative controls. The left histogram represents the microbiome composition for saline solution washed through a new bronchoscope; the right histogram represents the microbiome composition for saline solution washed through a reused sterilized bronchoscope. The sequencing libraries were prepared following the same protocol; thus, possible contaminations from reagents were also considered. Only genera with a read abundance higher than 1% in at least one sample are shown. Download FIG S4, TIF file, 0.1 MB. Copyright © 2018 Ren et al. 2018 Ren et al. This content is distributed under the terms of the Creative Commons Attribution 4.0 International license.

**FIG 5:**
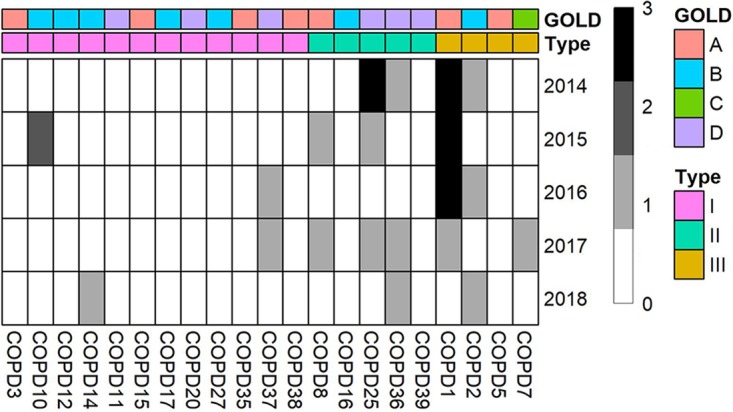
Exacerbation frequency in 21 COPD patients during 2014 to 2018. GOLD (Global Initiative for Obstructive Lung Disease) criteria were used to assess disease severity. A score of “A” represents the mild stage, and a score of “D” represents the most severe stage. Types were defined by microbial composition.

Although COPD is a chronic disease, some patients suffer from exacerbations. Recurrent exacerbation in COPD patients could lead to a faster decline in lung function and could increase their mortality risks. We have obtained the number of exacerbations for 21 COPD patients in the past 4 years (2014 to 2018) (after the bronchoscopy) (Fig. 5). In total, 29 exacerbation events were recorded; 14 of them occurred in 3 patients belonging to subgroup III, 10 of them occurred in 3 patients belonging to subgroup II, and 5 of them occurred in 3 patients belonging to subgroup I. The exacerbation frequency was significantly higher in patients belonging to subgroups II and III than in those belonging to subgroup I (2 and 3.5 versus 0.4, *P = *1.3e−5). This observation suggests that colonization of the lung by bacteria of some specific genera (e.g., *Streptococcus*, *Rothia*) might be protective against exacerbation whereas colonization by other bacteria (e.g., *Pseudomonas*) could be harmful. Interestingly, multiple studies have proposed that *Pseudomonas* could be a risk factor for exacerbation in COPD patients (24–26). Associations between individual microbes and clinical features are described in Text S1.

**FIG 6:**
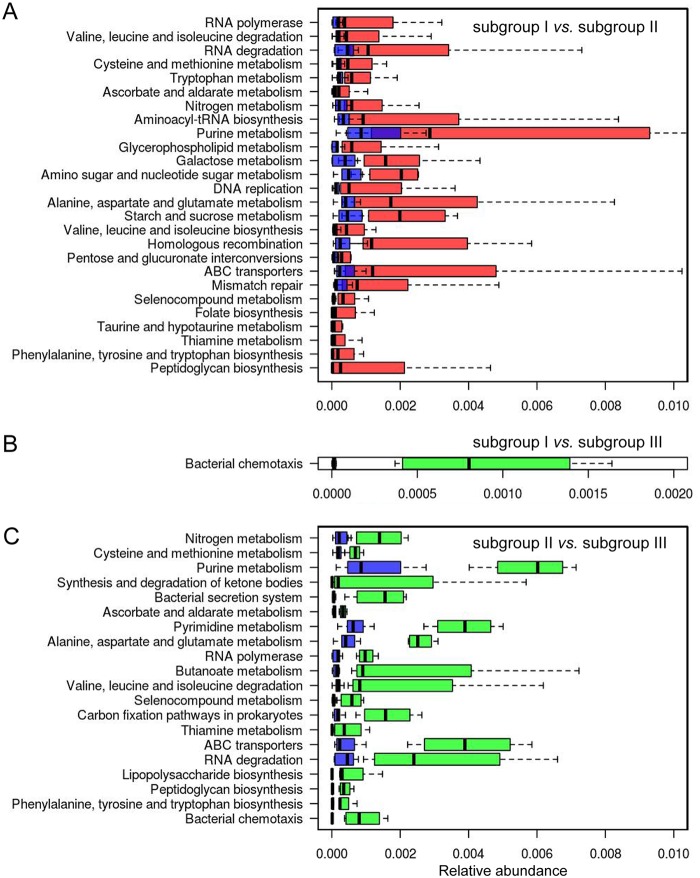
Enrichment of KEGG pathways in microbial genes in different samples. (A) Comparison between subgroup I and subgroup II. (B) Comparison between subgroup I and subgroup III. (C) Comparison between subgroup II and subgroup III. Only pathways with *P* values of <0.01 and *q* values of <0.1 (Mann-Whitney U test) are shown. The pathways were sorted by their fold changes in different subgroups (increasing from top to bottom). Red boxes represent subgroup I samples, blue boxes represent subgroup II samples, and green boxes represent subgroup III samples.

The highly transcribed microbial genes in subgroup I and III samples were similar and enriched for functional catalogs related to metabolism, biosynthesis, replication and repair, and membrane transport (Fig. 6).

### Interaction between active lung microbiome and host gene expression.

A large (>50%) proportion of the RNA reads in the BALF samples were actually derived from human cells (including macrophages, lymphocytes, and neutrophils) (27), enabling us to investigate the host-microbe interaction in 34 samples. We found that the expression levels of 10 genes were strongly correlated with the read abundance of specific microbes at the genus level (21 genes at the species level) (*P < *0.01 and *q *< 0.01) (Table S2); however, no specific biological or signaling pathway was enriched in the gene list.

10.1128/mSystems.00199-18.9 TABLE S2 Correlation between microbes and host gene expression. Download Table S2, XLSX file, 0.02 MB. Copyright © 2018 Ren et al. 2018 Ren et al. This content is distributed under the terms of the Creative Commons Attribution 4.0 International license.

**FIG 7:**
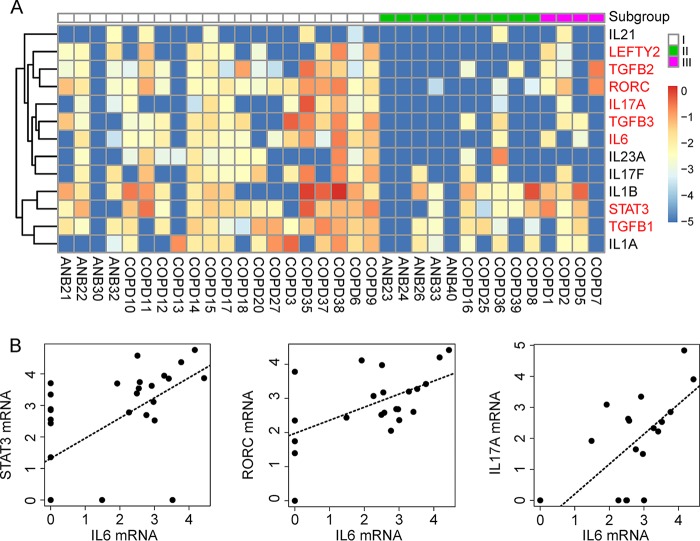
Activation of the Th17 cell differentiation pathway in humans. (A) Expression pattern of 13 key genes involved in Th17 cell differentiation. Differentially expressed genes are indicated in red (*P < *0.05). (B) Correlation between the expression of *IL-6* and downstream genes in the pathway (*STAT3*, *RORC*, and *IL17A*). The correlation coefficients (*rho*) were 0.641, 0.578, and 0.681, respectively (*P < *0.001). The gene expression level was calculated as log_2_(normalized number of transcripts per million [TPM] + 0.00001).

Thousands of genes were differentially expressed in the three microbiome subgroups (adjusted *P* value [*padj*], <0.01). Interestingly, one gene-enriched pathway (“differential regulation of cytokine production in macrophages and T helper cells by interleukin-17A [IL-17A] and IL-17F”) seems to be involved in the immune response to lung microbes, as CD4^+^ T helper (Th) cells can regulate the adaptive immune response against pathogens and their differentiation has been proposed to be associated with the lung microbiome (28–30). To further investigate the differentiation of Th cells, we compared the expression levels of 36 key genes in this pathway among the three subgroups (Fig. S5A). Overall, samples in subgroup I tended to have a higher expression level of all of these genes than samples in subgroup II (16 of them with *P < *0.05). In particular, the expression levels of the most critical molecules for Th17 cell differentiation (including IL-6, transforming growth factor β [TGFβ], STAT3, RORC, and IL-17) were all significantly increased in subgroup I samples, and these expression levels were highly synchronized (Fig. 7). The cytokine assay results further confirmed the increased levels of inflammatory cytokines (IL-6, IL-8, and IL-1α) in subgroup I samples (*P < *0.05, Fig. S5B) compared to those of subgroup II. Subgroup III is not discussed here due to the small sample size.

10.1128/mSystems.00199-18.6 FIG S5 Gene expression and cytokine levels in different subgroups. (A) Gene expression patterns of 36 key genes involved in Th cell differentiation. Gene expression levels were calculated as log_2_(normalized number of transcripts per million [TPM] + 0.00001). (B) Cytokine levels in different subgroup samples. Concentrations of 27 cytokine levels in the BALF were measured. Only cytokines whose concentrations differed among subgroups are shown (*P < *0.05). Download FIG S5, TIF file, 0.9 MB. Copyright © 2018 Ren et al. 2018 Ren et al. This content is distributed under the terms of the Creative Commons Attribution 4.0 International license.

We then looked for microbes that could potentially associate with the differentiation of Th17 cells. At the genus level, only *Gemella* was positively correlated with the expression of *IL-6* (Table S3). At the species level, seven species could potentially stimulate this process by upregulating key genes (Table S3).

10.1128/mSystems.00199-18.10 TABLE S3 Correlation between the expression of Th17 differentiation pathway genes and the read abundance of microbes. Download Table S3, XLSX file, 0.02 MB. Copyright © 2018 Ren et al. 2018 Ren et al. This content is distributed under the terms of the Creative Commons Attribution 4.0 International license.

## DISCUSSION

Although differences in lung microbiome between COPD and non-COPD samples have been found in several studies (15, 19–21), COPD is not the feature that explains most of the variance in lung microbiome in our study. Interestingly, we found that microbes enriched in COPD samples were mostly upper respiratory tract and oral (UO) microbes. They were found exclusively in 15 COPD samples (but were not correlated with severity of COPD), 12 of which had at least two UO microbes codetected (*P < *0.05). UO microbes can enter the lung through microaspiration but normally are quickly removed by the mucociliary clearance system in the lung. Such clearance is impaired in COPD patients (20, 31), and therefore enrichment of UO microbes is likely to be a consequence of COPD. This phenomenon is not specific to COPD but is also observed in other diseases, e.g., mechanically ventilated and pneumonia patients (31, 32).

The composition of the active lung microbiome observed in our study is similar to that found by other studies (2, 8, 9, 12, 33), except that two frequently observed high-abundance microbes, *Prevotella* and *Veillonella*, had relatively low read abundances in our study (1.4% and 3.6%). However, their abundances were higher in the 16S rRNA data (5.1% and 4.3%) and also much higher in the upper respiratory tract (throat swabs were available for seven samples) (see Fig. S6 in the supplemental material). We speculate that these two microbial genera in the lung were acquired by microaspiration from the upper respiratory tract and that their growth was likely to be suppressed by the regional conditions in the lung, although more upper respiratory tract samples were needed to address this issue.

10.1128/mSystems.00199-18.7 FIG S6 Read abundance changes between upper respiratory tract samples and lower respiratory tract samples. Five bacterial species with the biggest read abundance change are shown. The first column shows the read abundance in the throat swab, and the second column shows the read abundance in BALF for each bacterial species. Download FIG S6, TIF file, 0.1 MB. Copyright © 2018 Ren et al. 2018 Ren et al. This content is distributed under the terms of the Creative Commons Attribution 4.0 International license.

Since the discovery of enterotypes in the gut, similar microbial structures have been identified in other organs (34–36). The concept of a pneumotype was initially proposed by Segal and colleagues in healthy individuals; the researchers defined two pneumotypes according to the abundance of the oral microbes *Veillonella* and *Prevotella* in BALF samples (29, 33). Recently, Shenoy and colleagues also identified two pneumotypes (microbial community states) in HIV and pneumonia patients (30) but with different core microbes. Pneumotypes in both studies were identified on the basis of 16S rRNA data. In our study, three subgroups were identified from metatranscriptome data that may be more functionally relevant as they were inferred from the actively transcribed microbiome.

The subgroup I microbiome was dominated by *Streptococcus* and *Rothia* and was associated with high bacterial biomass, highly expressed microbial genes involved in metabolism and biosynthesis, and activation of the Th17 immune response. These features seem relevant, as bacterial growth requires an abundant nutrient supply and activation of microbial genes that absorb nutrients and synthesize proteins. Actively growing bacteria might activate the host defense system, including recruitment and differentiation of Th17 cells that mediate host defenses against microbes (37). Coincidentally, Vandeputte and colleagues have recently reported a similar association between microbial load and enterotype in gut (38). We speculate that the correlation could reflect the growth rate variation among different microbes under certain regional conditions. Moreover, COPD patients in this subgroup tended to experience less-frequent exacerbations, suggesting that bacterial colonization could be a crucial stimulus to airway inflammation and could thereby be a risk factor and represent a potential predictor of exacerbations in COPD patients.

Subgroup II had active *Escherichia* and *Ralstonia*, which have been discovered in the respiratory tract and mostly associate with pulmonary inflammation and cystic fibrosis (12, 39, 40). However, they could also have caused the contamination from reagents and bronchoscopes used in our study, as *Escherichia* was the most abundant component in the negative controls. Nevertheless, this subgroup is distinctive in terms of low bacterial biomass and should be considered separately.

Bacterial biomass and microbial gene function enrichment in subgroup III were similar to those in subgroup I, but the proportion of lymphocytes in subgroup III was much higher. This could indicate a more severe inflammation in this subgroup as lymphocytes are normally recruited into the alveolar space during inflammation (41); this may also associated with the fact that COPD patients in this subgroup experienced the most frequent exacerbations. Interestingly, an increased proportion of lymphocytes was found to be associated with the abundance of *Veillonella* (33). However, this correlation was not observed in our data (*P *> 0.05). Rather, the proportion of lymphocytes was found to be associated with the activity of Bordetella pertussis (Fig. 4F); infection with Bordetella pertussis is not rare in either the healthy population or COPD patients (42, 43), which could be related to the release of pertussis toxin (PT). PT could inhibit the recruitment of neutrophils and macrophages and could impede the movement of lymphocytes into lymph nodes (44). However, there are still samples that have a high proportion of lymphocytes but that have no Bordetella pertussis, suggesting that other mechanisms might be involved.

One limitation of the study was the relatively small number of microbial reads used in the analysis (median number = 8,276). Although a host rRNA depletion protocol was applied, 80% of the reads were derived from humans. On the one hand, this enabled us to investigate interactions between the microbiome and the host. On the other hand, less-active microbes could not be detected; however, rarefaction and variance analysis suggested that the data enabled us to identify the most active microbes and capture most of the variance among samples and thus should not have influenced the main conclusion of our study. Another limitation of our study was the relatively small sample size due to the difficulty encountered in collecting lower respiratory tract samples (as the procedure is invasive), which limited the statistical power of the study to detect differences between subgroups; thus, we focused only on the features that most closely associate with lung microbiome (bacterial biomass, Th17 immune response, COPD exacerbation frequency, etc.). Meanwhile, this potentially restricted the study to identification of only a subset of possible microbiome types. Actual lung microbiomes might be more diverse, and their association with clinical features could be more complicated. Nonetheless, data corresponding to the stratified structure of the transcriptionally active lung microbiome and its association with various features are all statistically significant and support the idea of an active host-microbe interaction. Together with previous studies on 16S rRNA and metagenome data, it is tempting to speculate that the lung microbiome variation is stratified in different dimensions in both healthy cases and some disease states. This stratification might represent differences in homeostasis states between the host and the microbiome. Critical follow-up studies should address to what extent the structures exist in different populations, how they are established and persist, and how they interact with the host immunity.

## MATERIALS AND METHODS

### Subjects and clinical samples.

Twenty-five COPD cases and nine non-COPD controls (not paired) were enrolled in this study. All COPD subjects were in a stable state (at least 8 weeks without exacerbation or use of antibiotics). The exclusion conditions included known cardiovascular diseases, renal or liver insufficiency, bronchiectasis, active pulmonary tuberculosis, bronchial asthma, pulmonary fibrosis, and lung cancer. Non-COPD controls had had no respiratory tract infection symptoms in the three months before submitting to bronchoscope examination. Clinical information was obtained for each enrolled patient (Table S1 in the supplemental material).

BALF samples were collected from each subject using a bronchoscope as part of normal clinical management. Two aliquots of 50 ml sterile isotonic saline solution were instilled, with 50% of the volume recovered on average. The BALF samples were immediately placed on ice and processed within 30 min. Bacterial culturing was performed on the BALF samples using an ATB Expression automatic bacterial identification instrument (bioMérieux, Marcy l’Etoile, France). The remnant samples were aliquoted and stored at −80°C before processing. Two negative controls (saline solution passed through a new bronchoscope and a reused sterilized bronchoscope) were collected and processed following the same library preparation protocol.

### Metatranscriptome sequencing.

A 1-ml aliquot of each whole-BALF sample was pretreated with Turbo DNase (Life Technologies, USA) to decrease the host genome background, according to the manufacturer's instructions. RNA was extracted using a QIAamp UCP pathogen minikit (Qiagen, Valencia, CA, USA), reverse transcribed, and amplified using an Ovation RNA-Seq system (NuGEN, CA, USA). Following fragmentation, the library was constructed using Ovation Ultralow System V2 (NuGEN, CA, USA) and was sequenced on an Illumina HiSeq 2500/4000 platform (Illumina, United Kingdom) (125-bp read length, paired-end protocol).

### Metatranscriptome data processing.

The raw data were first filtered by base quality score and read length using Trimmomatic (v0.35; SLIDINGINDOW:4:10 MINLEN:70) (45). All filtered reads that could be properly mapped to the human reference genome (GRCh38) or to human cDNA sequences (Ensembl release 83) by Bowtie2 (v2.2.6 –end-to-end, –sensitive) were suspected to represent host contamination and were discarded from further analysis (46). The remaining nonhuman reads were then searched against the ribosome RNA database using SortMeRNA (v2.1, –paired_out) (47), and the nonmapping reads were used for *de novo* assembly. Five assemblers were applied to the data, and the results were compared, “–pre_correction” was used for IDBA_UD and IDBA_Tran (48), “-k 31” was set for Ray(v2.3.1) (49), and default parameters were used for Trinity (v2.1.1) and SOAPdenovo2 (50, 51). Of note, none of these *de novo* assemblers performed well (see Text S1 in the supplemental material); thus, unassembled reads were used directly.

### Taxonomy assignment.

Unassembled reads were mapped against the NCBI nt database using BLASTN (v2.3.0, -task megablast, -evalue 1e-10, -max_target_seqs 10, -max_hsp 1 –qcov_hsp_perc 60) (52). The results were then used as the input for MEGAN 6 (Min Score 100, Top Percent: 10) (53), and the taxonomic assignment for each read was inferred using the lowest common ancestor (LCA) method. Meanwhile, nonhuman non-rRNA reads were also mapped to the NCBI nr database using Diamond (v0.7.11, –sensitive –c 1) (54), with the thresholds used in MEGAN6 modified accordingly (Min Score: 40, Max Expected 0.001). The conversion file from Gi number to KEGG was used to annotate the function of microbial reads (55). Unless stated otherwise, microbes with a read abundance of at least 1% (among all ABFV reads) in at least 1 sample were regarded as true positives and included in the analysis.

### 16S rRNA sequencing.

The V3-V4 hypervariable region of the bacterial 16S rRNA gene was amplified with barcoded primer set 341F (CCTAYGGGRBGCASCAG) and 806R (GGACTACNNGGGTATCTAAT) with an expected amplicon length of 466 bp. Sequencing of the amplicons was performed using an Illumina HiSeq 2500 instrument (Illumina, United Kingdom) (250-bp read length, paired-end protocol). Reads were analyzed by Mothur (v1.31.2) using the SILVA database (56, 57). Due to the low concentration of microbial DNA in the BALF, enough reads (>10,000) were obtained for only 20 samples with one repeat.

### Statistical analysis.

Pearson’s chi-square test or Fisher’s exact test was used for categorical variables, and the Mann-Whitney U test or Kruskal-Wallis rank sum test was used for continuous variables that do not follow a normal distribution. For multiple-test correction, the *q* value was calculated and a threshold value of 0.1 was applied (58). Benjamini and Hochberg’s adjusted *P* value (*padj*) was given by an integrated pathway analysis (IPA; Ingenuity Systems, Inc.) in the gene enrichment analysis (59), and a threshold value of 0.05 was applied.

More details of the methods employed are provided in the supplemental material.

### Ethics statement.

The study was approved by the Institutional Review Board of the Peking University People’s Hospital. All steps were carried out in accordance with relevant guidelines and regulations. Written informed consent was obtained from each participant.

### Data availability.

The metatranscriptome and 16S rRNA data have been submitted to NCBI's Sequence Read Archive (SRA) database under project number PRJNA390194.
