# Supplementary figures and images for: Transcriptionally Active Lung Microbiome and Its Association with Bacterial Biomass and Host Inflammatory Status
Source: mSystems. 2018 Oct 30;3(5):e00199-18. doi: 10.1128/mSystems.00199-18 (PMC6208642; doi:10.1128/mSystems.00199-18)

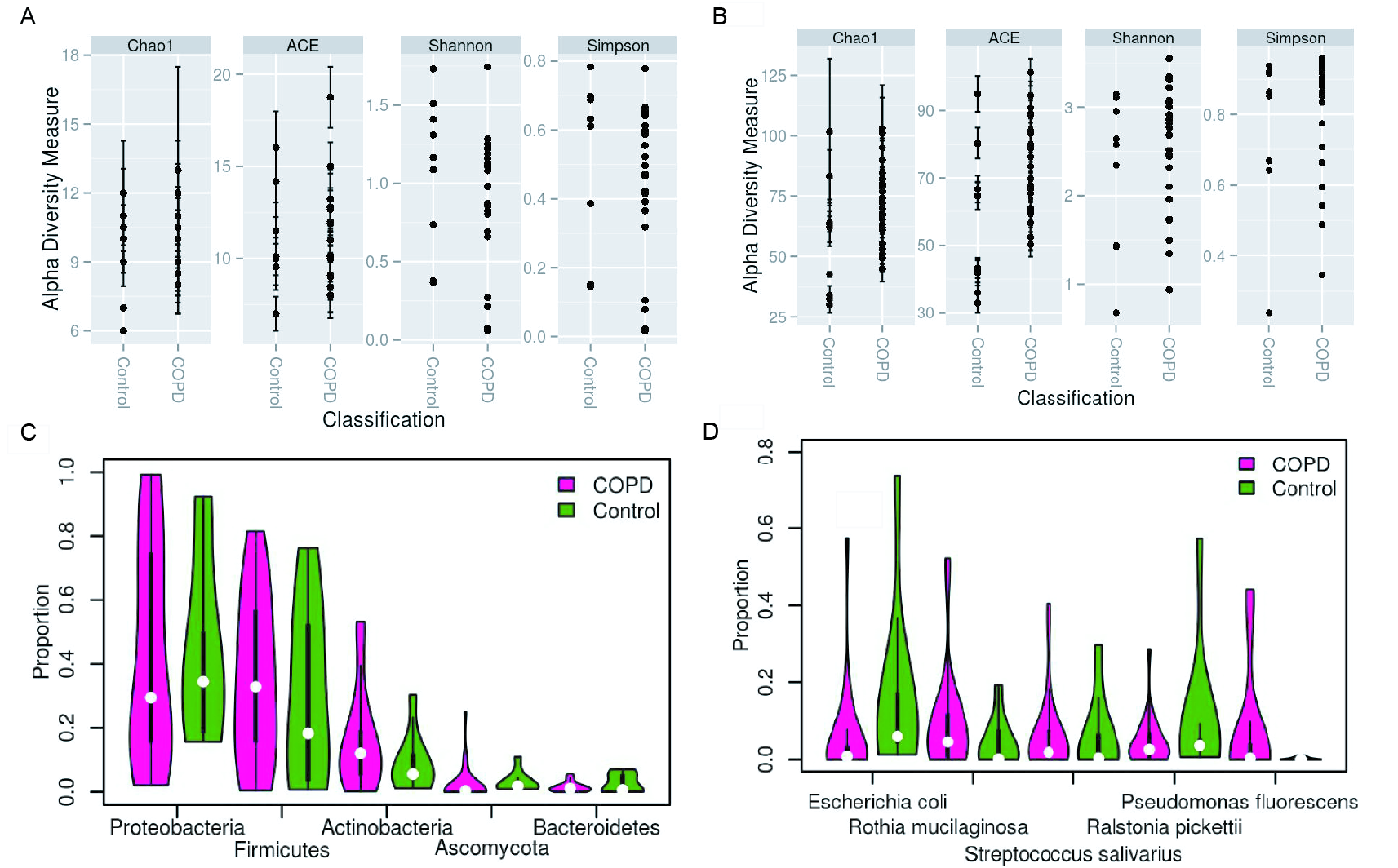

Supplement: FIG S1 [file sys006182285sf1.tif]

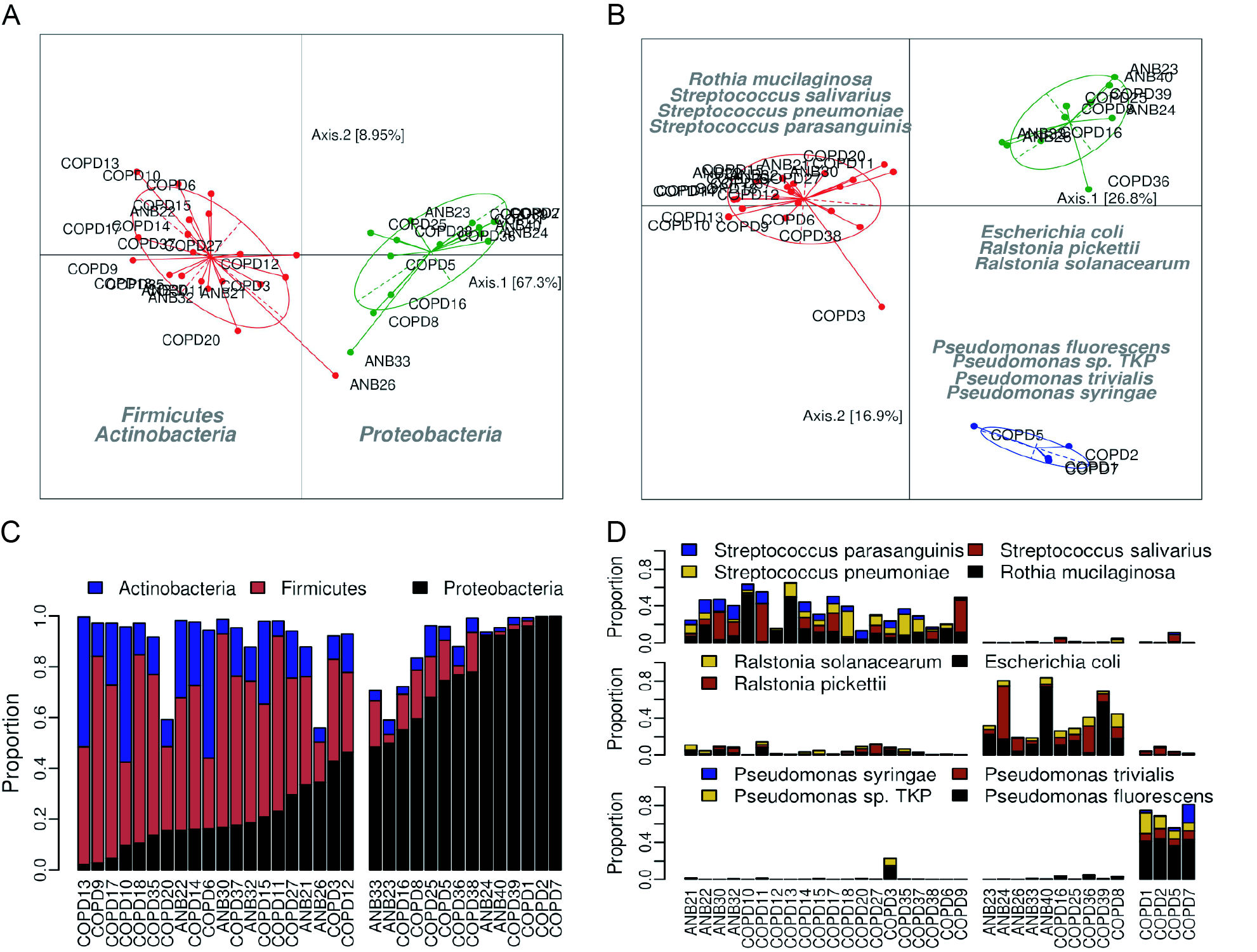

Supplement: FIG S2 [file sys006182285sf2.tif]

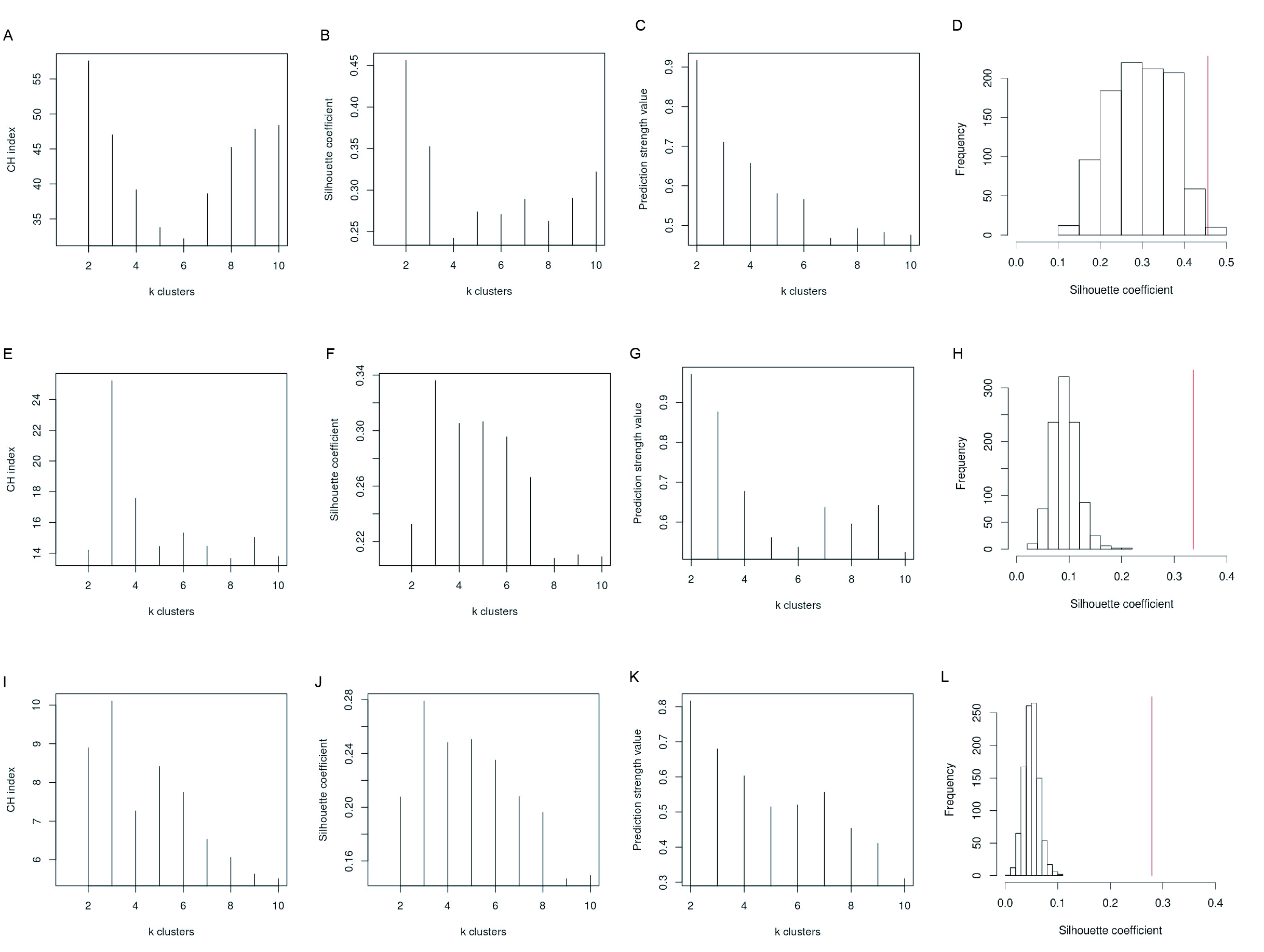

Supplement: FIG S3 [file sys006182285sf3.tif]

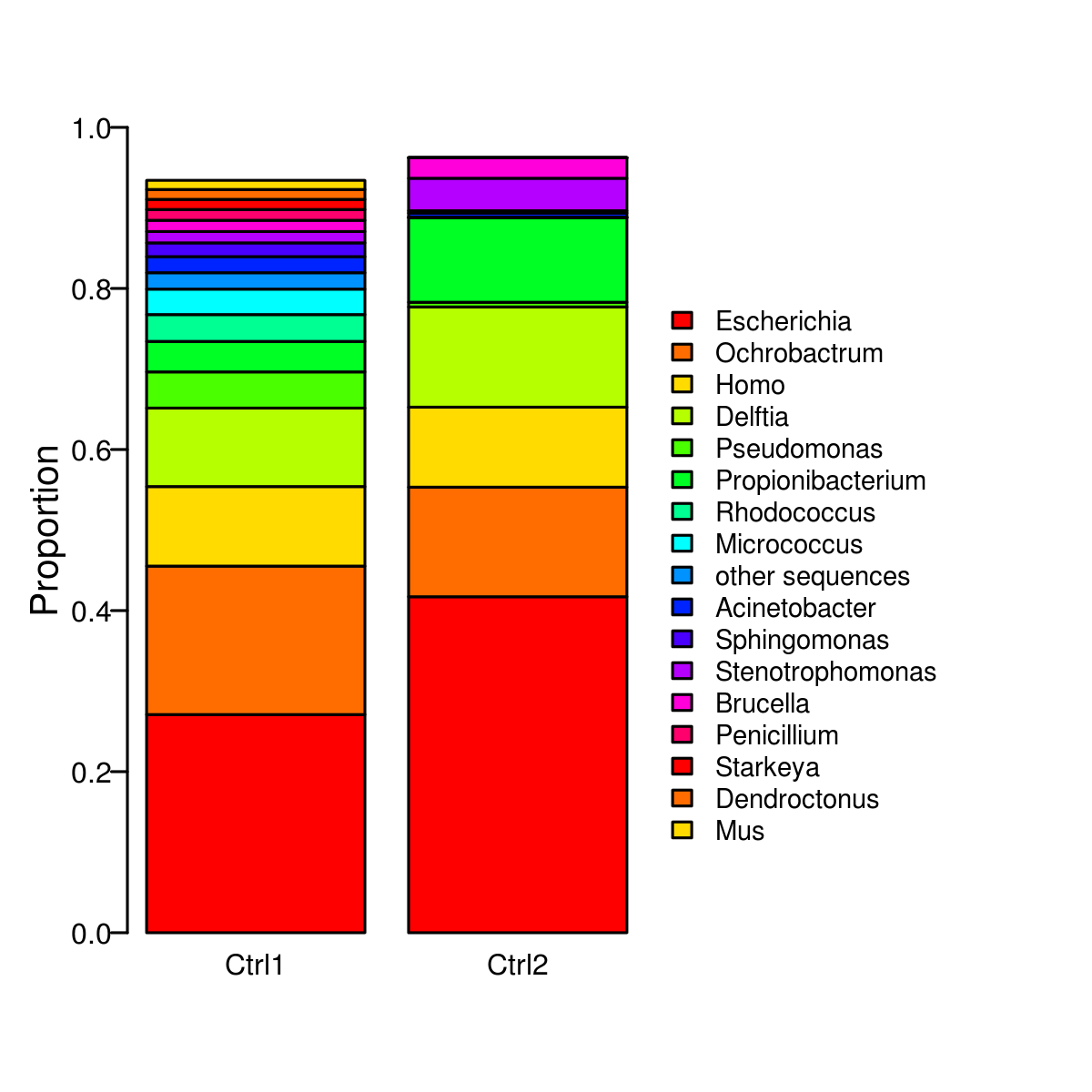

Supplement: FIG S4 [file sys006182285sf4.tif]

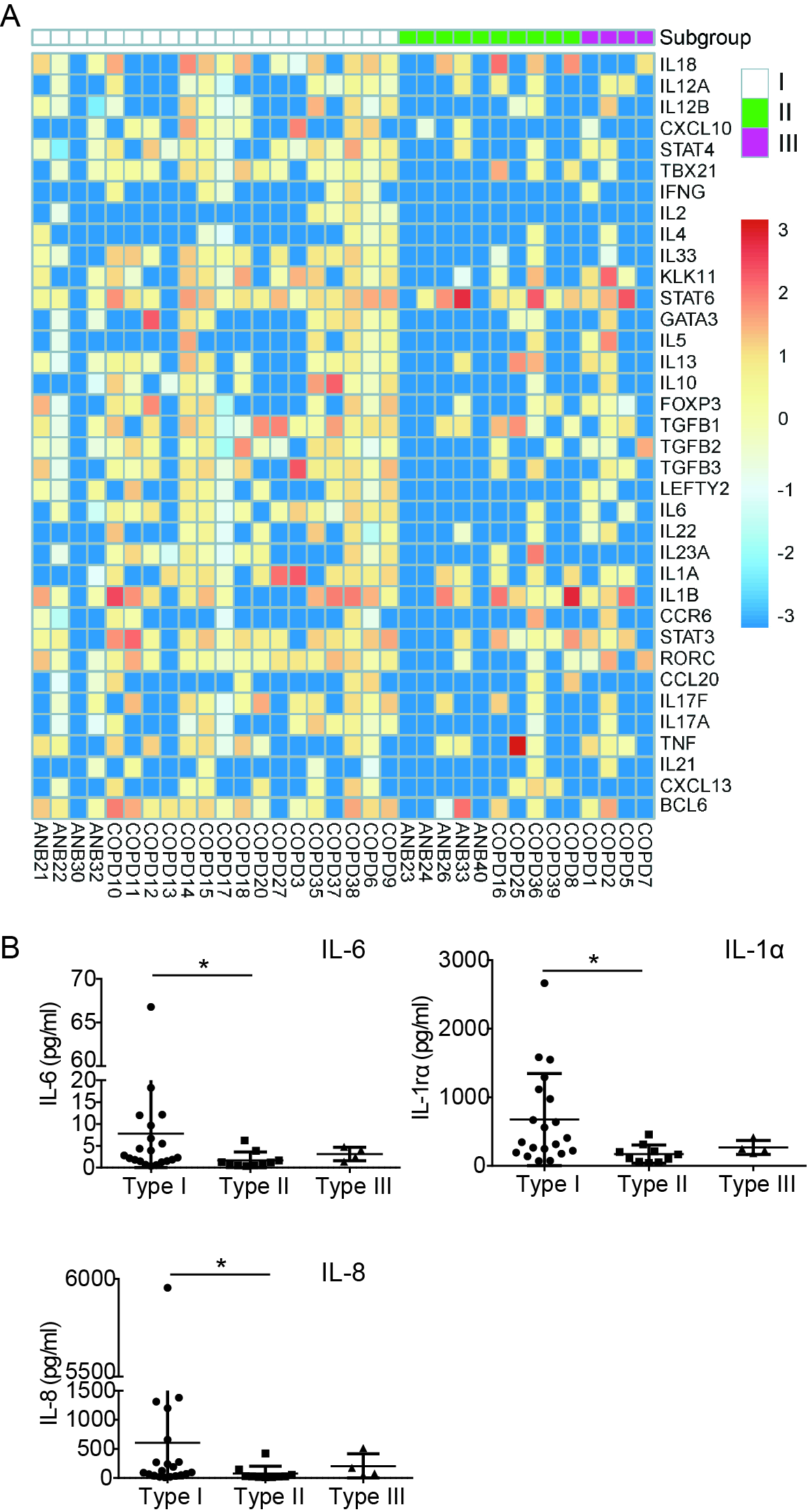

Supplement: FIG S5 [file sys006182285sf5.tif]

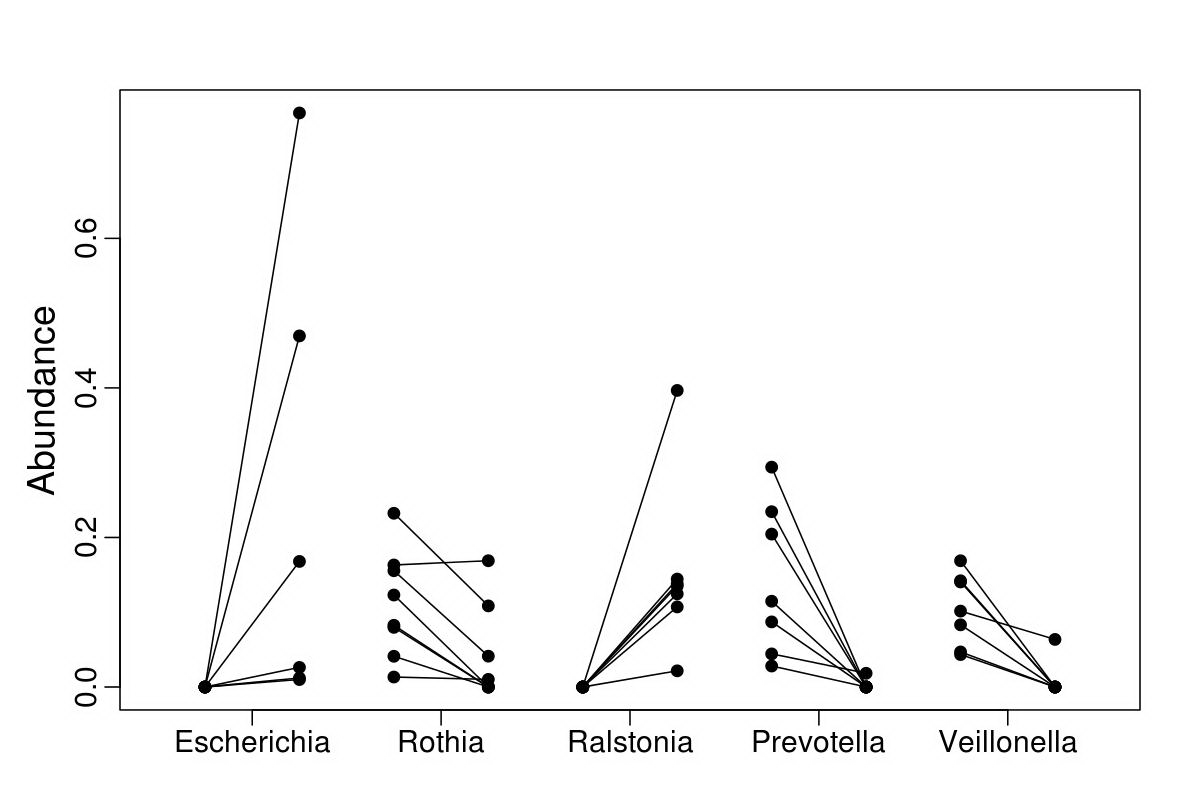

Supplement: FIG S6 [file sys006182285sf6.tif]
