# Supplementary material for: Transcriptionally Active Lung Microbiome and Its Association with Bacterial Biomass and Host Inflammatory Status
Source: mSystems. 2018 Oct 30;3(5):e00199-18. doi: 10.1128/mSystems.00199-18 (PMC6208642; doi:10.1128/mSystems.00199-18)
Supplement: TEXT S1 [file sys006182285s1.docx]

**Text S1. Supplemental Methods and Results:**

**Methods**

**Species identification**

The DNA sequence of species belonging to the same genus can be highly similar. Thus the number and length of species-specific sequences are highly variable for different species in a genus, leading to biased abundance estimation and unequal probabilities of identifying different species. To further confirm the existence of species identified by BLASTN, we selected all species represented by at least 100 reads in at least one sample as candidate species. We subsequently downloaded their reference genomes from the NCBI Genome/Assembly, and reads from all samples were merged and mapped to the genome of each species using Bowtie2 (--fast mode)(1). Candidate species were recognized as a true positive if at least 100 reads could be mapped to their genome, excluding rRNA or plasmid sequences. The abundance of each species (a) was calculated using the following formula:


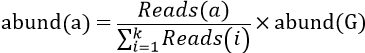


*Reads(a)* is the number of reads uniquely mapped to species *a*, *k* is the total number of species belonging to this genus, and *abund(G)* denotes the abundance of the genus.

**Human transcriptome analysis**

The high-quality reads were mapped to the human reference genome (GRCh38) by STAR (v2.0.4) with recommended parameters (--outFilterMultimapNmax 20 --alignIntronMin 20 --alignSJoverhangMin 8 --outFilterMismatchNmax 999 --alignSJDBoverhangMin 1 --alignIntronMax 1000000 --alignMatesGapMax 1000000) with the annotation file of GRCh38.84.gtf(2). Read counts and TPM (Transcripts per million) were calculated by StringTie (v1.2.3)(3), and TPM was further normalized by a housekeeping gene (*ACTB*). Differential expression analyses were performed using DESeq2 (v1.10.1)(4).

**Clustering based on the microbiome composition**

Two clustering methods were applied to investigate the underlying structure of the lung microbiome.

For the first strategy, pairwise distances, represented by Jensen–Shannon divergence (JSD) values, were calculated by the “distance” function implemented in the “phyloseq” R library(5, 6). The partitioning around medoids (PAM) algorithm was applied to cluster the abundance profiles based on the JSD matrix. As PAM is a supervised method, a series of cluster numbers (2-10) were given for the clustering procedure. The Calinski-Harabasz (CH) value was calculated by the “index.G1” function in the “clusterSim” R library; the cluster scheme with the highest CH value was chosen. Three statistical parameters were used to further evaluate the performance of clustering: 1) Average Silhouette Width (ASW), calculated by the “silhouette” function in the “cluster” R library (clustering with the highest ASW value should also have the highest CH value); 2) Predictive Strength (PS), calculated by the “prediction.strength” function in the “fpc” R package (an optimum clustering should have PS greater than 0.8); and 3) Simulation, for which 10,000 datasets with the same number of features (phylum/genus/species) and similar abundance of each feature were generated and the ASW was then calculated for each dataset and a *p*-value was calculated as the probability to have an ASW equal to or greater than the observed value. This clustering strategy was first introduced in two previous studies(7, 8), and has been adapted in our study.

For the second strategy, microbedmm was used for the Dirichlet Multinomial Mixtures analysis (9), and the cluster scheme with lowest Laplace approximation to the model evidence was chosen. Since the read count matrix was used as the input, the analysis was only applied to the phylum and genus data: species data was not used for this analysis because of the high number of unassigned reads (40%).

**Core microorganisms identification**

Three methods (Metastats, LEfSe, microbedmm) were applied to identify microbes that are differently distributed in two or more groups (9-11). The parameters are described as follows: *p*<0.05 and LDA>4.5 in LEfSe; *p*<0.05 and *q*<0.05 in Metastats (1,000 permutations); default parameters in microbedmm, only overlapped core microorganisms were regarded as potential core microorganisms. We further required a maximum *p-*value of 0.05 in Mann-Whitney tests (for two groups) or Kruskal-Wallis tests (for more than two groups), and core microorganisms have to be observed in all samples in the subgroup except at the species level.

**Bacteria DNA quantification**

The total bacterial DNA was quantified using the Femto^TM^ Bacterial DNA Quantification Kit (ZYMO Research, Irvine, CA) following the manufacturer’s protocol.

**Quantification of cytokines in BALF**

Twenty-seven cytokines were measured in the BALF using the Bio-PlexPro^TM^ Human Cytokine Standard 27-plex Group I (Bio-Rad), according to the manufacturer’s instructions, in a Bio-Rad 2000 system (Bio-Rad). The mean value of technical duplicates was used. The concentrations of IL-2, IL-5, IL-7, IL-10, and IL-13 were below the limit of detection and thus not included in the analyses.

**Results:**

**Data overview**

Approximately 8G of data (30 million paired-end reads) were generated on the Illumina HiSeq platform for each sample. After stringent quality control in terms of base quality and read length, 57% of the reads (range: 32%-69%) could be properly mapped to the human reference genome in their full length. Although rRNA was not preferentially enriched, 5% of the reads mapped to the rRNA database. Half of the non-human non-rRNA reads could be mapped to the NCBI nt database while only 6% of the reads could be mapped to the NCBI nr database. Although five assemblers (IDBA-Tran, IDBA-UD, Trinity, Ray, SOAPdenovo) were applied to perform the de-novo assembly, none of these could achieve good performance. The average length of contig and N50 of the assembly were both shorter than 200bp and the proportion of singleton reads that could not map to any contig (>=100bp) were more than 80% in some individuals. Thus, unassembled reads were used directly.

The NCBI nt database was used as a reference for Megablast. The taxonomy assignment was determined by the Lowest Common Ancestor (LCA) method implemented in MAGEN. Most reads (98.6%) were assigned to the *Metazoa* kingdom, where 92% of such reads could be further assigned to *Homo sapiens*. This suggests they are potentially the human reads from regions that are too diverse to be mapped to the human reference genome with their full length in the previous step. The human RNA accounts for 76.7% (±11.5%) of the total RNA. After removing the reads assigned to *Metazoa* and *Viridiplantae*, 99.3% of the remaining reads were assigned to Archaea, Bacteria, Fungi and Viruses (ABFV). These are likely the true composition of the lower respiratory tract microbial community. The proportion of ABFV reads ranges from 0.003% to 0.867% (with median of 0.02%), while the absolute number ranges from 1272 to 411217 (with median of 8276) in different samples. Notably, 92% of the microbial reads could be assigned to a specific genus, and 60% could be assigned to a specific species or subspecies.

**Association between lung microorganisms and clinical features**

In addition to investigating the association between microbes and COPD, we further looked into the correlation between the abundance of microbes and various clinical features. Across all three taxonomic levels, we found two significant associations after multiple testing correction. First, there is a negative correlation between the abundance of the genus *Alphacoronavirus* and the patients’ CAT (COPD Assessment Test) score (*rho*=-0.725, *p*<0.001, *q*<0.05). The latter is an index used to quantify the impact of COPD symptoms, where a higher CAT score indicates a worse scenario (12). The second correlation is between the abundance of the species *Rothia dentocariosa* and age (*rho*=0.634, *p*<0.001, *q*<0.05). This indicates that older individuals tend to have a higher abundance of the oral microbe *Rothia dentocariosa* in their lower respiratory tract. Since these features are not independent and could correlate with many other factors (e.g., whether older people tend to have more *Rothia dentocariosa* in their mouth), the association needs to be validated in a better matched case–control study.

**References**

1. Langmead B, Salzberg SL. Fast gapped-read alignment with Bowtie 2. Nature methods. 2012;9(4):357-9.

2. Dobin A, Davis CA, Schlesinger F, Drenkow J, Zaleski C, Jha S, et al. STAR: ultrafast universal RNA-seq aligner. Bioinformatics (Oxford, England). 2013;29(1):15-21.

3. Pertea M, Pertea GM, Antonescu CM, Chang TC, Mendell JT, Salzberg SL. StringTie enables improved reconstruction of a transcriptome from RNA-seq reads. Nature biotechnology. 2015;33(3):290-5.

4. Love MI, Huber W, Anders S. Moderated estimation of fold change and dispersion for RNA-seq data with DESeq2. Genome biology. 2014;15(12):550.

5. McMurdie PJ, Holmes S. phyloseq: an R package for reproducible interactive analysis and graphics of microbiome census data. PloS one. 2013;8(4):e61217.

6. R Core Team (2013). R: A language and environment for statistical computing. R Foundation for Statistical Computing, Vienna, Austria. URL <http://www.R-project.org/>.

7. Arumugam M, Raes J, Pelletier E, Le Paslier D, Yamada T, Mende DR, et al. Enterotypes of the human gut microbiome. Nature. 2011;473(7346):174-80.

8. Tyakht AV, Kostryukova ES, Popenko AS, Belenikin MS, Pavlenko AV, Larin AK, et al. Human gut microbiota community structures in urban and rural populations in Russia. Nature communications. 2013;4:2469.

9. Holmes I, Harris K, Quince C. Dirichlet multinomial mixtures: generative models for microbial metagenomics. PloS one. 2012;7(2):e30126.

10. Segata N, Izard J, Waldron L, Gevers D, Miropolsky L, Garrett WS, et al. Metagenomic biomarker discovery and explanation. Genome biology. 2011;12(6):R60.

11. White JR, Nagarajan N, Pop M. Statistical methods for detecting differentially abundant features in clinical metagenomic samples. PLoS computational biology. 2009;5(4):e1000352.

12. Lee SD, Huang MS, Kang J, Lin CH, Park MJ, Oh YM, et al. The COPD assessment test (CAT) assists prediction of COPD exacerbations in high-risk patients. Respiratory medicine. 2014;108(4):600-8.
